# Rifampin resistance and multidrug resistance or extensive drug resistance (MDR/XDR) predict failure in monomicrobial *Staphylococcus epidermidis* periprosthetic joint infection (PJI) – a 15-year single-center cohort study

**DOI:** 10.5194/jbji-11-237-2026

**Published:** 2026-04-21

**Authors:** Álvaro Auñon, Santiago Gabardo, Carmen Álvaro, Ricardo de la Concha, Javier Sanado, Jaime Esteban

**Affiliations:** 1 Department of Orthopaedic Surgery, Hospital Universitario Fundación Jiménez Díaz, Madrid, Spain; 2 Networking Research Centre on Infectious Diseases, Madrid, Spain; 3 Department of Internal Medicine, Hospital Universitario Fundación Jiménez Díaz, Madrid, Spain; 4 Department of Clinical Microbiology, Hospital Universitario Fundación Jiménez Díaz, Madrid, Spain

## Abstract

**Introduction:** *Staphylococcus epidermidis* is a leading coagulase-negative pathogen in periprosthetic joint infection (PJI), but the outcome impact of key resistance phenotypes is unclear. **Methods:** We retrospectively studied consecutive monomicrobial *S. epidermidis* PJIs treated at a tertiary hospital (2010–2024). PJI was defined according to 2018 ICM criteria. Success required infection eradication without suppressive antibiotics or further infection-related surgery after ≥ 12 months. **Results:** Among 516 evaluable PJIs, 105 (20.3 %) were monomicrobial PJIs due to *S. epidermidis*. The mean age was 71, and 56 % of cases were women; 51 % of cases involved the knee, and 49 % involved the hip. Chronic infection accounted for 79 %. Surgical strategies were DAIR in 27.6 % of cases, one-stage revision in 28.6 % of cases, and two-stage revision in 39.0 % of cases. Overall success was 78.1 % (DAIR – 75.9 %, one-stage – 93.3 %, two-stage – 73.2 %). Resistance rates were as follows: methicillin – 72.3 %, fluoroquinolone – 53.3 %, rifampin – 24.8 %, and multidrug resistance or extensive drug resistance (MDR/XDR) – 43.8 %. Rifampin resistance (73.1 % vs. 91.3 %, p = 0.021) and MDR/XDR (67.4 % vs 84.7 %, p = 0.035) were associated with failure; methicillin ( p = 0.853) and fluoroquinolone resistance ( p = 0.129) were not. In the univariable analyses, levofloxacin resistance was significantly associated with treatment failure (OR (odds ratio): 3.71; 95 % CI (confidence interval): 1.58–8.69; p = 0.002), as was rifampicin resistance (OR: 5.83; 95 % CI: 2.22–15.34; p < 0.001). Double resistance to levofloxacin and rifampicin showed the strongest association with failure (OR: 8.01; 95 % CI: 2.82–22.77; p < 0.001). **Conclusion:** Rifampin-resistant and MDR/XDR *S. epidermidis* PJIs represent higher-risk infections and should prompt early optimization of biofilm-active therapy and surgical source control. Univariable analysis confirmed rifampin resistance; levofloxacin resistance; and, in particular, their combination as strong predictors of treatment failure.

## 1 Introduction

Periprosthetic joint infection (PJI) is one of the most significant complications after total hip and knee arthroplasty and remains a leading cause of failure and revision surgery. Although its incidence after primary arthroplasty is relatively low, with an incidence ranging from approximately 1.5 % to 2.5 % of cases, the growing number of primary and revision procedures means that the absolute burden of PJI is steadily increasing (Flurin et al., 2019). These infections lead to repeated operations, prolonged antibiotic therapy, impaired function, and a substantial consumption of healthcare resources (Marculescu and Cantey, 2008; Tan et al., 2016; Wimmer et al., 2016).

Staphylococci are the predominant pathogens in PJI, and, within this group, coagulase-negative staphylococci (CoNS), especially *Staphylococcus epidermidis*, have emerged as key etiological agents in hip and knee PJI, accounting for up to 23 %–25 % of the cases in several studies (Lange et al., 2025). Traditionally regarded to be low-virulence commensals, *S. epidermidis* strains have adapted remarkably well to the prosthetic joint environment. Their ability to adhere to biomaterials and to form biofilm favors persistence on the implant surface and substantially compromises the effectiveness of systemic antimicrobial therapy (Rohde et al., 2010).

When treating PJI it is important to consider planktonic bacteria, especially relevant during the initial phases of acute infections (Ariza et al., 2017). Initial therapy should target rapidly growing planktonic bacteria, typically with β -lactams, lipopeptides, or glycopeptides, followed by orally bioavailable agents with good bone penetration combined with drugs active against biofilm-embedded bacteria, such as rifampin (Auñón et al., 2022). Accordingly, characterization of antimicrobial susceptibility – particularly to β -lactams, fluoroquinolones, and rifampin – is essential in *S. epidermidis* PJI as resistance to these agents significantly impacts treatment strategy and outcomes.

Methicillin resistance is common in *S. epidermidis* PJI, reflecting hospital antibiotic pressure and the organism's capacity to accumulate resistance determinants (reported in 18 %–45 % of isolates) (Jiang et al., 2025; Lange et al., 2025). Methicillin resistance (MRSE) limits β -lactam options and may affect early management, while fluoroquinolone and rifampin resistance further compromise standard oral and biofilm-active regimens (Hischebeth et al., 2019).

A similar situation has been described for fluoroquinolone resistance. Several studies have identified the absence of an active fluoroquinolone-based regimen as a factor associated with treatment failure in *Staphylococcus aureus* prosthetic joint infection; nonetheless, these analyses were not specifically focused on coagulase-negative staphylococci, and results were reported for *S. aureus* rather than for CoNS species as a distinct group (Lora-Tamayo et al., 2013). Several authors have reported poorer outcomes in staphylococcal prosthetic joint infection caused by rifampin-resistant isolates. In the landmark study by Zimmerli et al. (1998), which included *S. aureus* and other staphylococcal species, the absence of rifampin activity was associated with significantly higher rates of treatment failure, establishing rifampin as a major determinant of prognosis in implant-associated staphylococcal infections. Similarly, Tornero et al. (2014) analyzed risk factors for failure in early prosthetic joint infection managed with debridement, antibiotics, and implant retention, identifying rifampin resistance as the main treatment-related factor associated with failure among gram-positive microorganisms overall. However, again, these analyses did not specifically focus on CoNS.

The aim of the present study is to characterize prosthetic joint infections caused by *S. epidermidis* at our institution by describing their epidemiology, clinical presentation, microbiological features, surgical and antimicrobial management, and outcomes.

## 2 Material and methods

### 2.1 Study design and setting

A retrospective observational study was conducted in a third-level university hospital, including all consecutive patients diagnosed with monomicrobial *S. epidermidis* PJI between January 2010 and December 2024. Data were collected from electronic medical records and analyzed in compliance with institutional ethical guidelines. The study was approved by our institution's ethics committee (reference no. EO128-20_FJD).

### 2.2 Data collection

Demographic variables such as age, gender, American Society of Anesthesiologists (ASA) classification, and Charlson Comorbidity Index (CCI) were recorded. Additional patient-related factors included body mass index (BMI), smoking habit, alcohol consumption, and presence of diabetes mellitus or immunosuppression, as well as chronic obstructive pulmonary disease, chronic kidney disease, and heart disease. Regarding surgical data, the joint affected (hip or knee), nature of the index surgery (primary or revision), and subsequent surgical interventions were documented in detail. The interval between the index arthroplasty and infection onset was also registered to categorize infections as acute ( ≤ 4 weeks from index surgery) or chronic ( > 4 weeks from index surgery). Hematogenous prosthetic joint infection (PJI) was defined as the occurrence of infection in a previously well-functioning and asymptomatic prosthetic joint, associated with either the identification of a distant infectious source responsible for the bacteremia or the acute onset of joint-related symptoms in the absence of criteria fulfilling a primary (perioperative or contiguous) PJI.

### 2.3 Diagnostic workup and case definition

All patients underwent a standardized preoperative diagnostic protocol. This included routine blood tests (white blood cell count, erythrocyte sedimentation rate, and C-reactive protein); radiographic evaluation; and, when feasible, joint aspiration for microbiological and cytological analysis. Synovial fluid was analyzed for leukocyte count and percentage of polymorphonuclear cells. Intraoperative tissue and fluid samples were systematically collected and sent for microbiological analysis. The diagnosis of PJI was established according to the 2018 International Consensus Meeting criteria (Parvizi et al., 2018) following preoperative tests such as white blood cell counts; erythrocyte sedimentation rate; C-reactive protein measurements; and, when feasible, joint aspiration and microbiological cultures from five to seven samples. The date of diagnosis was defined as the date of the initial surgical intervention when positive cultures were obtained. Cases diagnosed prior to 2018 were retrospectively classified according to the 2018 ICM criteria.

### 2.4 Microbiological methods

Samples were processed according to our institutional protocol, previously described (Prieto-Borja et al., 2018). Periprosthetic tissues were homogenized, and removed implants underwent sonication in sterile containers with phosphate buffer. Sonicate fluid was centrifuged, and the resulting concentrate was inoculated onto aerobic and anaerobic culture media. Culture plates were incubated according to routine laboratory practice, generally for up to 14 d, although some media required shorter incubation periods.

Bacterial identification was performed using MALDI-ToF mass spectrometry once this technology became available at our institution (2014); prior to that, identification was based on conventional biochemical techniques.

Antimicrobial susceptibility was determined mainly through automated testing systems, with additional manual methods being used when needed. Results were interpreted following EUCAST recommendations. Monomicrobial *S. epidermidis* PJI was defined as recovery of *S. epidermidis* as the only microorganism from periprosthetic samples that met ICM diagnostic criteria. Microorganisms were classified according to resistance profiles: no resistance, resistance to rifampin, resistance to quinolones, resistance to oxacillin, multidrug resistance (MDR), and extensive drug resistance (XDR) as defined by Magiorakos et al. (2012).

### 2.5 Antimicrobial susceptibility testing and resistance definitions

The antimicrobial susceptibility testing of the organisms was performed using an automated system (VITEK. 2, Biomerieux) and, in some cases, Etest and disk-plate assays. The tested antibiotics were those included in the VITEK. cards and, for manual methods, those recommended for specific microorganisms. We considered rifampin and quinolones as the key antibiotics for the treatment of gram-positive PJI, as previous articles have demonstrated in the literature (Magiorakos et al., 2012). Because rifampin plus a fluoroquinolone represents the most established oral biofilm-active combination for staphylococcal implant-associated infection, we performed an additional clinically oriented analysis stratifying isolates according to combined rifampin and fluoroquinolone susceptibility. Moreover, in vitro data suggest that both antimicrobials are the most active ones against staphylococcal biofilms, and so this approach looks for the impact of these resistances on the patients' outcomes.

#### 2.5.1 Multidisciplinary management and treatment strategy

Cases were discussed weekly by a multidisciplinary infection team including orthopedic surgeons, infectious disease specialists, and microbiologists. Treatment was individualized, with patients undergoing DAIR, one-stage revision, or two-stage revision, as well as salvage procedures reserved for failures. Decisions followed institutional protocols with allowance for clinical discretion.

Antimicrobial therapy was tailored to culture results and administered according to international guidelines. Intravenous antibiotics were typically initiated immediately postoperatively and continued for a minimum of 2 to 6 weeks, followed by oral therapy when indicated. Antimicrobial duration and changes were recorded in the patient file.

### 2.6 Inclusion and exclusion criteria

A minimum follow-up period of 12 months from the final surgical intervention was required for inclusion. Patients with less than 12 months of follow-up or those lost to follow-up were excluded from the final analysis. Loss to follow-up was defined as the absence of any clinical visit or laboratory evaluation during the 12-month period after definitive treatment. Furthermore, patients with polymicrobial periprosthetic joint infection (PJI) were excluded from the analysis.

### 2.7 Outcome definitions

The primary endpoint was treatment success, defined as eradication of infection with no clinical signs, no need for suppressive antibiotics, and no additional surgery, according to the MusculoSkeletal Infection Society (MSIS) (Fillingham et al., 2019). Treatment failure included persistent or recurrent infection, the need for further surgical procedures, or infection-related mortality.

### 2.8 Statistical analysis

Continuous variables were summarized as means, and categorical variables were summarized as percentages. Baseline characteristics were compared using chi-square or Fisher's exact tests for categorical variables and Student's t test for continuous variables, with p<0.05 considered to be significant. Univariable analyses were performed to identify factors associated with treatment success, calculating odds ratios (ORs) with 95 % confidence intervals (CIs). Firth's penalized logistic regression was applied when appropriate, and analyses were conducted using R software (R Foundation for Statistical Computing, Vienna, Austria).

## 3 Results

A total of 615 patients diagnosed with periprosthetic joint infection (PJI) were identified and initially considered for inclusion in the present study. After applying exclusion criteria, 105 cases (20.35 %) were caused by *S. epidermidis*.

The mean age of the cohort was 71 years old (SD: 10.4). Females accounted for 56 % of patients. The knee joint was the most frequently involved site, accounting for 51 % of PJIs, while the hip was affected in 49 % of cases.

When considering the type of implant involved, 59 % of infections occurred in primary prosthetic implants, and 41 % occurred in revision arthroplasties. A total of 83 patients (79 %) met the chronic infection definition, whereas 22 (20 %) were diagnosed as acute infection.

From a surgical risk perspective, the mean ASA Score was 2.49; furthermore, the mean CCI score was 3.73. Assessment of BMI showed that 45.7 % of the study population suffer from obesity (BMI ≥ 30 kg m^−2^).

As for related risk factors, 14.3 % of the patients had diabetes mellitus. Demographic data and comorbidity profiles are shown in Table 1.

**Table 1:** Baseline demographics of the study population.

Risk factor	
Mean age (years old)	70.4
Sex (% female/male)	56/44
Joint (knee/hip)	51/49
ASA	2.49
CCI score	3.73
Obesity (%)	45.7 (48)
Diabetes (%)	14.3 (15)
Immunosuppression (%)	3.8 (4)
COPD (%)	14.1 (15)
Heart disease (%)	17.1 (18)
Chronic kidney disease (%)	2.8 (3)

In terms of surgery, 29 patients underwent DAIR ( 29/105 , 27.6 %), 30 underwent one-stage revision ( 30/105 , 28.6 %), 41 underwent two-stage revision ( 41/105 , 39.0 %), and 5 underwent other procedures ( 5/105 , 4.8 %) such as resection arthroplasty or arthrodesis. Surgical data are shown in Table 2 for further reference.

**Table 2:** Surgical data of the study population.

Global success rate (%)	78.1 (82)
Acute infection (%)	20.9 (22)
Chronic infection (%)	79.1 (83)
DAIR (%)	27.6 (29)
One-stage revision (%)	28.6 (30)
Two-stage revision (%)	39 (41)
DAIR success rate (%)	75.9 (23)
One-stage revision success rate (%)	93.3 (28)
Two-stage revision success rate (%)	73.1 (30)

Overall, treatment was successful in 82 patients ( 82/105 , 78.1 %). Between the surgical procedures, the success rates were 75.9 % for DAIR, 93.3 % for one-stage revision, and 73.1 % for two-stage revision. None of the recorded demographic characteristics or risk factors were significantly associated with treatment failure. Similarly, neither MDR/XDR infection nor rifampin resistance was significantly associated with any of the collected baseline variables. Of the 23 failures, 10 (43.5 %) were due to persistent infection, and 9 (39.1 %) were due to superinfection. Superinfections were most frequently caused by Enterobacteriaceae (44.5 %), followed by *Proteus mirabilis* (33.3 %), *Klebsiella pneumoniae* (11.1 %), and *Candida albicans* (11.1 %).

The mean duration of antimicrobial therapy was 10.1 weeks overall. Patients treated with DAIR received longer courses (mean: 11.8 weeks) compared with those undergoing one-stage revision (10.6 weeks). In contrast, patients managed with two-stage revision had a shorter mean treatment duration (8.5 weeks).

### 3.1 Methicillin-resistant *S. epidermidis*

Among the 105 monomicrobial *S. epidermidis* PJIs, 76 isolates (72.3 %) were methicillin resistant (MRSE). In the MRSE group, the mean age was 70.8 years (range: 53–88); 40 patients were female (52.6 %); and the knee was the most frequently involved joint, with 45 cases (59.2 %) occurring after total knee arthroplasty. The mean ASA class was 2.47, and the mean CCI score was 3.86. The most common comorbidities were obesity (45.4 %), chronic obstructive pulmonary disease (15.8 %), and diabetes mellitus (14.5 %).

Regarding infection chronicity, 60 patients (79.0 %) were classified as having chronic PJI, and 16 (21 %) were classified as having as acute PJI. None of these baseline demographic, clinical, or infection-related variables differed significantly compared with the methicillin-susceptible group. Data are shown in Table 3.

At final follow-up, 59 of 76 MRSE patients achieved 77.6 % treatment success, comparable to the methicillin-susceptible group (79.3 %), without statistically significant differences ( p=0.853 ).

**Table 3:** Data from the methicillin-susceptible Staphylococcus epidermidis (MSSE) and MSRE cohort.

	MSSE ( n=29 )	MRSE ( n=76 )	p
Mean age (years old)	71.4	70.8	0.356
Sex	65.5/35.5	52.6/47.4	0.237
Joint (knee/hip)	58.6/41.4	59.2/40.8	0.867
ASA	2.55	2.47	0.612
CCI score	4.03	3.86	0.511
Obesity (%)	44.8 (13)	45.4 (34)	0.905
Diabetes (%)	13.8 (4)	14.5 (11)	0.929
Immunosuppression (%)	3.5 (1)	4 (3)	0.904
COPD (%)	10.3 (3)	14.4 (11)	0.577
Heart disease (%)	17.4 (5)	14.4 (11)	0.724
Chronic kidney disease (%)	0 (0)	5.2 (4)	0.208
Acute infection	6	16	0.967
Chronic infection	23	60	0.967
Success rate	79.3	77.6	0.853
Mean antibiotic duration (weeks)	11.15	9.77	0.296

### 3.2 Fluoroquinolone-resistant *S. epidermidis*

A total of 56 out of the 105 (53.3 %) *S. epidermidis* isolated were fluoroquinolone resistant (FRSE). In this subgroup, the mean age was 71.7 years (range: 53–87), and 29 patients (51.8 %) were women. Most infections involved the knee, with 36 cases (64.3 %) following total knee arthroplasty. The average ASA class was 2.46, and the mean CCI score was 4.12. Obesity was the most frequent comorbidity (46.4 %), followed by heart disease (17.9 %) and chronic obstructive pulmonary disease (16 %) and diabetes mellitus (12.5 %).

Based on the time from index surgery, 42 patients (75.0 %) were categorized as having chronic PJI, and 14 (25.0 %) were categorized as having acute PJI. No significant differences were observed in terms of baseline characteristics or infection chronicity compared with the fluoroquinolone-susceptible group. Data are shown in Table 4.

By the end of follow-up, treatment success was achieved in 41 of 56 fluoroquinolone-resistant cases (73.2 %). Although the fluoroquinolone-susceptible infection treatment success rate was higher (85.4 %), this difference did not reach statistical significance ( p=0.1288 ).

**Table 4:** Data from the fluoroquinolone-susceptible Staphylococcus epidermidis (FSSE) and FSRE cohort.

	FSSE ( n=49 )	FRSE ( n=56 )	p
Mean age (years old)	70.2	71.7	0.451
Sex	61.2/38.8	51.8/48.2	0.431
Joint (knee/hip)	53.1/46.9	64.3/35.7	0.272
ASA	2.53	2.46	0.521
CCI score	3.65	4.12	0.470
Obesity (%)	44.9 (22)	46.4 (26)	0.320
Diabetes (%)	16.3 (8)	12.5 (7)	0.572
Immunosuppression (%)	2 (1)	5.3 (3)	0.3758
COPD (%)	12.2 (6)	16 (9)	0.576
Heart disease (%)	16.3 (8)	17.9 (10)	0.835
Chronic kidney disease (%)	0 (0)	7.1 (4)	0.056
Acute infection	7	14	0.1709
Chronic infection	42	42	0.1709
Success rate (%)	85.4	73.2	0.1288
Mean antibiotic duration (weeks)	10.17	9.98	0.877

### 3.3 Rifampin-resistant *S. epidermidis*

Rifampin resistance was identified in 26 of 105 monomicrobial *S. epidermidis* PJIs (24.8 %). In this rifampin-resistant subgroup (RRSE), the mean age was 71 years (range: 49–83), and 18 patients were women (69.2 %). The knee was the most commonly affected joint, with 17 cases (58.6 %) occurring after total knee arthroplasty. The mean ASA class was 2.38, and the mean CCI score was 4.11. The most prevalent comorbidities were obesity (46.1 %), chronic obstructive pulmonary disease (15.4 %), and diabetes mellitus (15.4 %).

Most RRSE infections were chronic ( 22/26 , 84.6 %), with the remaining four cases (15.4 %) being classified as acute. Baseline demographic and clinical characteristics did not differ significantly from those of the rifampin-susceptible group. Data are shown in Table 5.

Interestingly, treatment success in this group was lower than in rifampin-susceptible infections (73.1 % vs. 91.3 %), with this difference being statistically significant ( p=0.021 ).

**Table 5:** Data from the rifampin-susceptible Staphylococcus epidermidis (RSSE) and RRSE cohort.

	RSSE ( n=79 )	RRSE ( n=26 )	p
Mean age (years old)	71	71	0.999
Sex	51.9/48.1	69.2/30.8	0.191
Joint (knee/hip)	54.4/45.6	58.6/41.4	0.871
ASA	2.53	2.38	0.422
CCI score	3.83	4.11	0.541
Obesity (%)	45.6 (36)	46.1 (12)	0.958
Diabetes (%)	13.9 (11)	15.4 (4)	0.872
Immunosuppression (%)	2.5 (2)	7.7 (2)	0.233
COPD (%)	13.9 (11)	15.4 (4)	0.853
Heart disease (%)	20.2 (16)	7.7 (2)	0.111
Chronic kidney disease (%)	2.5 (2)	7.7 (2)	0.269
Acute infection	7	4	0.346
Chronic infection	72	22	0.346
Success rate (%)	91.3	73.1	0.021
Mean antibiotic duration (weeks)	11.72	11.25	0.290

### 3.4 MDR/XDR* S. epidermidis*

MDR/XDR profiles were observed in 46 of 105 monomicrobial *S. epidermidis* PJIs (43.8 %). Patients in this subgroup had a mean age of 70.2 years (range 44–88), and 26 were female (56.2 %). The knee was the predominant site of infection, with 32 cases (69.6 %) occurring after total knee arthroplasty. The mean ASA class was 2.48, and the mean CCI score was 4.09. The most frequent comorbidities were obesity (47.8 %), chronic obstructive pulmonary disease (15.2 %), and heart disease (13 %).

With respect to timing, most MDR/XDR cases were chronic ( 35/46 , 76.1 %), while 11 (23.9 %) were acute. No significant differences in terms of baseline demographic or clinical variables were identified compared with non-MDR/XDR infections. Data are shown in Table 6.

At final follow-up, 31 of 46 MDR/XDR infections met the criteria for treatment success (67.4 %). This was significantly lower than the success rate observed in susceptible isolates (84.7 %; p=0.035 ).

**Table 6:** Data from the susceptible/MDR/XDR cohort.

	Susceptible ( n=59 )	MDR/XDR ( n=46 )	p	
Mean age (years old)	71.6	70.2	0.753	
Sex	55.9/43.1	56.2/43.8	0.989	
Joint (knee/hip)	50.8/49.2	69.6/30.4	0.123	
ASA	2.51	2.48	0.546	
CCI score	3.76	4.09	0.999	
Obesity (%)	44.1 (26)	47.8 (22)	0.931	
Diabetes (%)	16.9 (10)	10.9 (5)	0.263	
Immunosuppression (%)	1.7 (1)	6.5 (3)	0.404	
COPD (%)	15.3 (9)	15.2 (7)	0.225	
Heart disease (%)	18.6 (11)	13 (6)	0.363	
Chronic kidney disease (%)	5.1 (3)	2.2 (1)	0.404	
Acute infection	9	9	0.491	
Chronic infection	50	35	0.491	
Success rate (%)	84.7	67.4	0.035	
Mean antibiotic duration (weeks)	10.39	9.71	0.581	

### 3.5 Univariable analysis

In univariate analysis, resistance to key antibiotics was significantly associated with failure. Levofloxacin resistance was associated with increased odds of treatment failure (OR: 3.71; 95 % CI: 1.58–8.69; p=0.002 ), as was rifampicin resistance (OR: 5.83; 95 % CI: 2.22–15.34; p<0 .001). Notably, double resistance to both levofloxacin and rifampicin demonstrated the strongest association with failure (OR: 8.01; 95 % CI: 2.82–22.77; p<0 .001). Conversely, oxacillin resistance was not associated with failure of treatment (OR: 0.90; 95 % CI: 0.70–1.55; p=0.156 ), suggesting that loss of both components of a standard biofilm-active oral strategy may be more prognostically relevant than resistance to methicillin per se. Data are shown in Table 7.

**Table 7:** Data from the univariable analysis.

	OR	IC 95 %	p
Levofloxacin-resistant	3.71	1.58–8.69	0.002
Rifampin-resistant	5.83	2.22–15.34	< 0.001
Methicilin-resistant	0.90	0.70–1.55	0.156
Levofloxacin- and rifampin-resistant	8.01	2.82–22.77	< 0.001

## 4 Discussion

In our single-center cohort of 105 monomicrobial *S. epidermidis* PJIs, the overall treatment success rate was 78.1 %. The most clinically relevant findings were that rifampin resistance and MDR/XDR phenotypes were each associated with significantly lower success, whereas methicillin resistance and fluoroquinolone resistance were not statistically associated with failure in this series.

The proportion of PJIs caused by *S. epidermidis* (20.3 %) in our cohort is consistent with contemporary epidemiology, in which CoNS and *S. epidermidis* represent a major fraction of causative organisms. Large institutional series and reviews commonly report *S. epidermidis* rates around the mid-20 % range among PJI pathogens, supporting the fact that our microbiological distribution is representative of current practice (Jiang et al., 2025; Lange et al., 2025)

A high prevalence of resistant pathogens was observed at 72.3 % MRSE, 53.3 % fluoroquinolone-resistant, 24.8 % rifampin-resistant, and 43.8 % MDR/XDR, reflecting the growing prevalence of multidrug resistance in PJI (Lange et al., 2025).

We observed the highest success with one-stage revision (93.3 %), followed by DAIR (75.9 %) and two-stage revision (73.1 %). This could likely reflect a selection bias effect rather than intrinsic superiority of one-stage exchange. In many centers, two-stage revision is preferentially used for more complex infections, which can depress success rates. However, the favorable results of the one-stage approach in our cohort are consistent with European practice, where careful patient selection and a feasible targeted antimicrobial plan support its effectiveness (Lange et al., 2025).

Conversely, implant retention strategies can perform poorly in CoNS infections, particularly in unfavorable microbiological settings. In one cohort of CoNS knee PJI, the overall 1-year success rate was 47 %, and outcomes after DAIR were especially low (Charalambous et al., 2022).  The relatively favorable DAIR success observed in our cohort should be interpreted cautiously. DAIR outcomes depend heavily on timing, implant stability, adequacy of debridement, exchange of modular components, host factors, and – especially in staphylococcal infections – the availability of an active biofilm-oriented regimen. Available evidence suggests that patients in whom rifampin can be used tend to achieve higher DAIR success rates, with cure rates around 70 % compared to approximately 50 % in those not receiving rifampin (Scheper et al., 2021).

Our findings support the fact that, in *S. epidermidis* PJI, especially when resistance limits effective antibiotic regimens, adequate source control through implant removal may be critical to achieve infection eradication.

Successful management of staphylococcal PJI requires effective anti-biofilm combination therapy beyond activity against planktonic bacteria. The randomized trial by Zimmerli et al. demonstrated superior outcomes with rifampin-based regimens in implant-related infections treated with debridement (Zimmerli et al., 1998). This benefit is reflected in guideline recommendations. SEIMC guidance highlights rifampin as the key agent against biofilm-associated staphylococci, particularly in DAIR, and recommends rifampin–fluoroquinolone combinations as a preferred oral regimen when feasible (Lora-Tamayo et al., 2013).

Within this framework, our finding that rifampin-resistant *S. epidermidis* had significantly worse outcomes is both clinically coherent and relevant. Rifampin resistance eliminates one of the few consistently supported oral anti-biofilm strategies and forces reliance on rifampin-sparing regimens, for which evidence is less robust and tolerability can be limiting (Lora-Tamayo et al., 2013; Zimmerli et al., 1998). Where rifampin cannot be used, alternative combinations (e.g., tetracycline-based strategies such as minocycline-containing regimens) have been proposed, but supporting data are comparatively limited and often non-randomized (Bart et al., 2020).

MDR/XDR phenotypes were also associated with failure, likely through multiple mechanisms. These profiles limit the feasibility of constructing effective, biofilm-active, and well-tolerated regimens and may reflect prior antibiotic exposure or more complex clinical scenarios. Moreover, MDR frequently coexists with rifampin and fluoroquinolone resistance, further restricting optimal consolidation strategies (Magiorakos et al., 2012). In our series, resistance to rifampin and levofloxacin in combination showed the strongest association with treatment failure, with an odds ratio of approximately 8.

In contrast to rifampin resistance and MDR/XDR, methicillin resistance was not associated with worse outcomes in our cohort, despite reports from two-stage revision series showing lower eradication rates in MRSE compared with methicillin-susceptible infections (Hischebeth et al., 2019). Similarly, two-stage revision for methicillin-resistant hip PJI has been reported, with infection control rates around 71 % (methicillin-resistant) vs. ∼ 90 % (controls), highlighting the potential adverse impact of resistant organisms, even in exchange settings (Santoso et al., 2020). This discrepancy likely reflects differences in terms of case mix and surgical approach rather than the absence of a biological disadvantage for MRSE as methicillin resistance alone may be less impactful when adequate source control and effective therapy are achieved. Fluoroquinolone resistance showed a trend toward lower success, although without statistical significance, possibly due to limited power or alternative regimens. However, because rifampin-based strategies commonly rely on a fluoroquinolone partner, this resistance pattern remains clinically relevant and may have a greater prognostic impact in larger or DAIR-focused cohorts (Lora-Tamayo et al., 2013; Zimmerli et al., 1998).

Overall, our findings suggest that outcomes in *S. epidermidis* PJI depend largely on the feasibility of administering an effective biofilm-active regimen, often determined by rifampin susceptibility. In the presence of rifampin resistance or MDR/XDR profiles, implant exchange may be preferable to DAIR, given the greater reliance of retention strategies on optimal antimicrobial therapy (Charalambous et al., 2022; Lora-Tamayo et al., 2013, 2013).

The practical implication of our findings is that antimicrobial resistance should influence not only antibiotic selection but also the surgical strategy. In susceptible isolates, particularly when rifampin and a suitable partner drug remain available, implant-retention strategies may still be reasonable in selected acute cases. By contrast, when rifampin resistance or combined rifampin–fluoroquinolone resistance is present, the feasibility of a standard oral biofilm-active regimen is substantially reduced. In those scenarios, surgeons and infection specialists should have a lower threshold for implant exchange, particularly when other unfavorable features coexist, such as chronic infection, compromised soft tissues, multiple prior procedures, or limited modular exchange. In this setting, the use of new antimicrobials with high antibiofilm activity (like linezolid, tedizolid, dalbavancin, and others) can be used according to the individualized antimicrobial susceptibility of each strain and the specific characteristics of the patients (Valour et al., 2025)

Finally, emerging anti-biofilm strategies could become particularly relevant in resistant *S. epidermidis* PJI, where traditional treatment options are often limited. There is increasing focus on approaches such as bacteriophage therapy, phage–antibiotic combinations, biofilm-disrupting agents, and alternative drugs with potential anti-biofilm activity. However, the evidence remains limited and is mostly based on experimental ground, small series, or selected compassionate-use cases. For now, these strategies should be used as adjuncts rather than as a replacement for established surgical and antimicrobial approaches (Ortiz-Cartagena et al., 2025).

Despite being one of the largest single-center series dedicated to monomicrobial *S. epidermidis* PJI, this study's retrospective design and long study period introduce potential confounding, and surgical comparisons are subject to selection bias. Additionally, resistance profiles were used as surrogates for treatment feasibility, and detailed regimen-level analyses would better clarify their impact on outcomes.

## 5 Conclusions

In this cohort of monomicrobial *S. epidermidis* PJI, rifampin resistance and MDR/XDR phenotypes were associated with significantly poorer outcomes, reinforcing the clinical importance of maintaining access to biofilm-active combination therapy and of aligning surgical strategy with microbiological “treatability”. The strong performance of one-stage exchange in selected patients suggests that, when source control is adequate and antimicrobial options are favorable, high success is achievable, even in a population with a substantial antimicrobial resistance pattern.

## Data Availability

The data of this study are not publicly available due to patient confidentiality requirements and institutional data protection regulations but are available from the corresponding author upon reasonable request.
